# Evaluation of pilot implementation of seasonal malaria chemoprevention on morbidity in young children in Northern Sahelian Ghana

**DOI:** 10.1186/s12936-021-03974-x

**Published:** 2021-11-18

**Authors:** Patrick O. Ansah, Nana A. Ansah, Keziah Malm, Dennis Awuni, Nana Peprah, Sylvester Dassah, Sobe Yarig, Charles Manful, John Agbenyeri, John Awoonor-Williams, Wilfred Ofosu, Abraham R. Oduro

**Affiliations:** 1 Navrongo Health Research Centre, Research and Development Division, Ghana Health Service, Navrongo, Ghana; 2 Public Health Division, National Malaria Control Programme, Ghana Health Service, Accra, Ghana; 3 Upper East Regional Health Directorate, Ghana Health Service, Bolgatanga, Ghana; 4 Northern Regional Health Directorate, Ghana Health Service, Tamale, Ghana; 5 Upper West Regional Health Directorate, Ghana Health Service, Wa, Ghana

**Keywords:** Feasibility, Seasonal-malaria-chemoprevention, Children, Northern Ghana

## Abstract

**Background:**

In Sahelian Africa, the risk of malaria increases with the arrival of the rains, particularly in young children. Following successful trials, the World Health Organization (WHO) recommended the use of seasonal malaria chemoprevention (SMC) in areas with seasonal peak in malaria cases. This study evaluated the pilot implementation of SMC in Northern Ghana.

**Methods:**

Fourteen communities each serving as clusters were selected randomly from Lawra District of Upper West Region as intervention area and West Mamprusi District in the Northern Region as the non-intervention area. The intervention was undertaken by the National Malaria Control Programme in collaboration with regional health directorates using sulfadoxine-pyrimethamine plus amodiaquine and standard WHO protocols. Before and after surveys for malaria parasitaemia and haemoglobin levels as well as monitoring for malaria morbidity and mortality were undertaken.

**Results:**

At the end of the intervention, participant retention was 92.9% (697/731) and 89.5% (634/708) in the intervention and the non-intervention areas, respectively. The proportion of children with asexual parasites reduced by 19% (p = 0.000) in the intervention and increased by 12% (p = 0.000) in the non-intervention area. Incidence rates of severe malaria were 10 and 20 per 1000 person-years follow up in the intervention and comparison areas, respectively with P.E of 45% (p = 0.62). For mild malaria, it was 220 and 170 per 1000 person-years in intervention and comparison area, respectively with PE of - 25% (p = 0.31). The proportion of children with anaemia defined as Hb< 11.0 g/dl reduced from 14.2% (52.8–38.6%) in the intervention area as compared to an increase of 8.1% (54.5% to 62.6) the non-intervention arm, Mean Hb reduced by 0. 24 g/dl (p = 0.000) in the non-intervention area and increased of 0.39 g/dl (p = 000) in the intervention area.

**Conclusions:**

The feasibility and effectiveness of SMC introduction in Northern Ghana was demonstrated as evidenced by high study retention, reduction in malaria parasitaemia and anaemia during the wet season.

## Background

Malaria remains a major public health concern in Sub-Saharan Africa (SSA) despite a significant reduction in the global burden of the disease in the last two decades [1]. Sub-Saharan Africa still accounts for most clinical cases and malaria-related deaths, which contributes about 10% of all under-five deaths. Malaria exerts negative social and economic impact in endemic areas. Economically it reduces productivity due to worker absenteeism and increased national health care spending. Socially, malaria affects individual and household behaviours resulting in broad social costs with the attendant long-term effect on growth and development [1, 2].

Currently, the World Health Organization (WHO) recommends a combination of approaches for malaria control including the use of long-lasting insecticide-treated nets (ITNs), indoor residual spraying (IRS), confirmation and use of artemisinin-based combination treatment (ACT) for case management [1, 2]. In addition, intermittent preventive treatment in pregnancy (IPTp) and children (IPTc) are recommended for specific high-risk groups [1, 2]. The IPTc has been found to be a safe method of malaria control that reduces significant proportion of clinical malaria illness in areas of marked seasonal malaria transmission. It also has substantial protective effect against all-cause mortality. Therefore, IPTc as valuable tool can contribute to the control of malaria especially in children in areas with short and marked seasonal transmission [3].

The WHO currently recommends Seasonal Malaria Chemoprevention (SMC) as a tool to prevent malaria among young children in areas with high seasonal transmission especially in the Sahelian areas of Africa [4, 5]. The SMC is the intermittent administration of full malaria treatment course to children during the malaria season. This is to check infection by sustaining therapeutic concentrations of anti-malarial drugs in the blood during the period of highest malarial risk [4, 5]. The WHO recommends sulfadoxine-pyrimethamine plus amodiaquine as the drugs of choice in the Sahel sub-region of Africa, where *Plasmodium falciparum* is sensitive to both anti-malarial drugs [4, 5]. Across the Sahel sub-region, most childhood malarial disease and deaths occur during the rainy season, which is generally short (3–4 months). Giving effective anti-malarial treatment at monthly intervals during this period protects against uncomplicated and severe malaria in young children [6–8].

The SMC approach is cost-effective and safe, and can be administered by community-health workers. In areas where SMC has been adopted it has helped in reducing the cases of malaria and proven to be very effective [6–8]. Apart from reducing infection, morbidity and mortality, malaria anaemia and child wellbeing are also improved [6–8]. Several studies in different countries attest that SMC is very effective in the fight against malaria and should be scaled up in other areas that had more cases of malaria [6–8].

Despite the success, the SMC strategy has some challenges. The implementation and acceptance of treatment drugs have been an issue for some time. The issue of implementation particularly the last mile distribution across hard-to-reach areas have often emerged. Also, studies have shown that some mothers do not administer the anti-malarial medication as prescribed. Parents give several reasons for not adhering to the medication, such as the drugs being bitter and complaints of the negative side effects they get from these drugs. Despite this, there is good adherence to the SMC drugs in most areas where it has been implemented, and care givers have attested to them being beneficial by helping them in reducing the incident cases of malaria [9, 10].

The Ghana National Malaria Control Programme (NMCP) in 2014 proposed to adopt SMC using the sulfadoxine-pyrimethamine plus amodiaquine combination in Ghana [11]. The adoption was to help reduce morbidity and mortality in children in the northern Ghana where malaria transmission is seasonal. This was to also improve the wellbeing and the livelihoods of many children and parents as time and resources are spent to cater for malaria that could be channeled into other productive ventures. However, there was a need for the NMCP to undertake a pilot study to determine the feasibility and effectiveness of SMC in the Ghanaian setting to inform policy decision for its scale-up.

## Methods

### Study setting

**Fig. 1:**
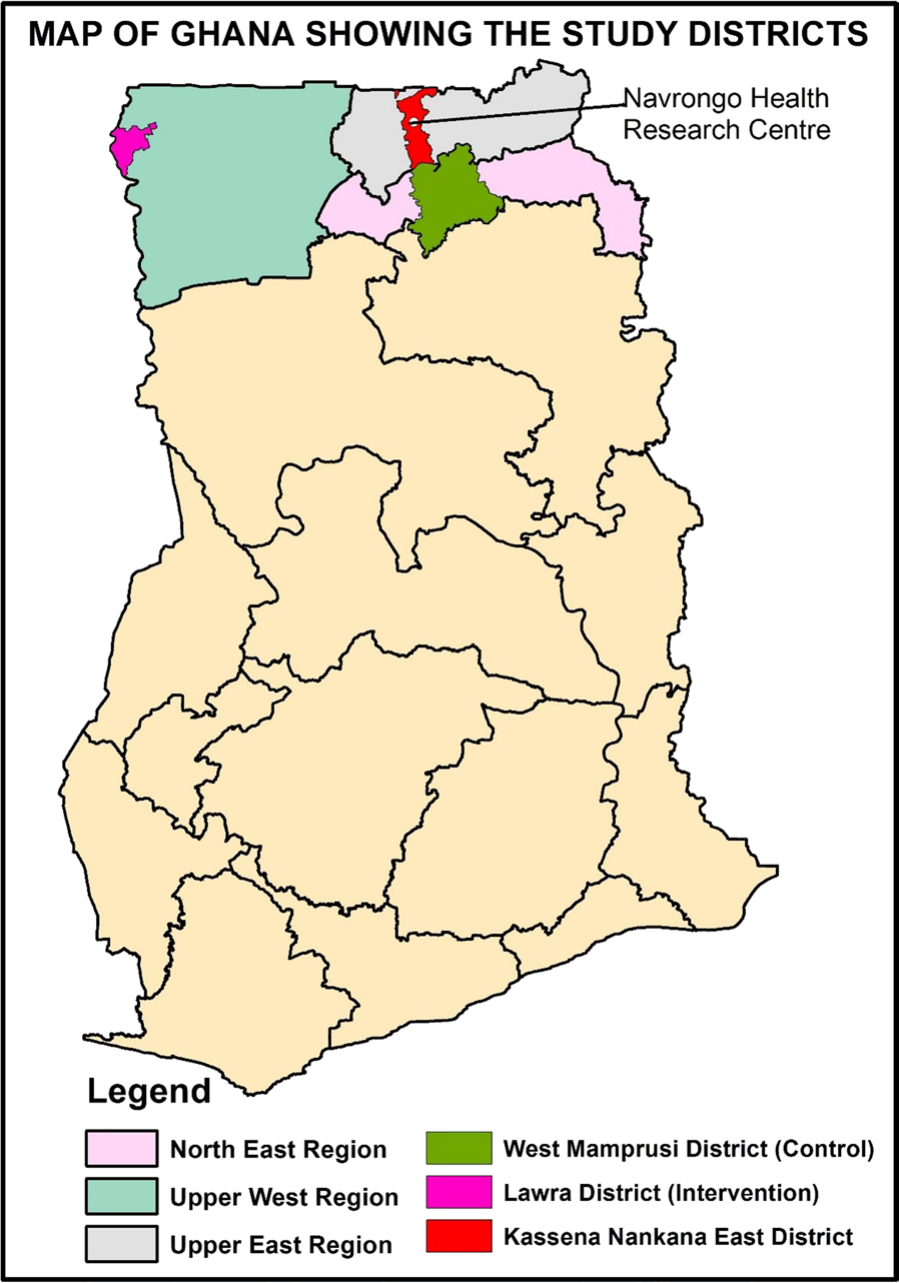
Map of Ghana showing the regions and districts of the intervention and control areas and that of the Navrongo Health Research centre

**Fig. 2:**
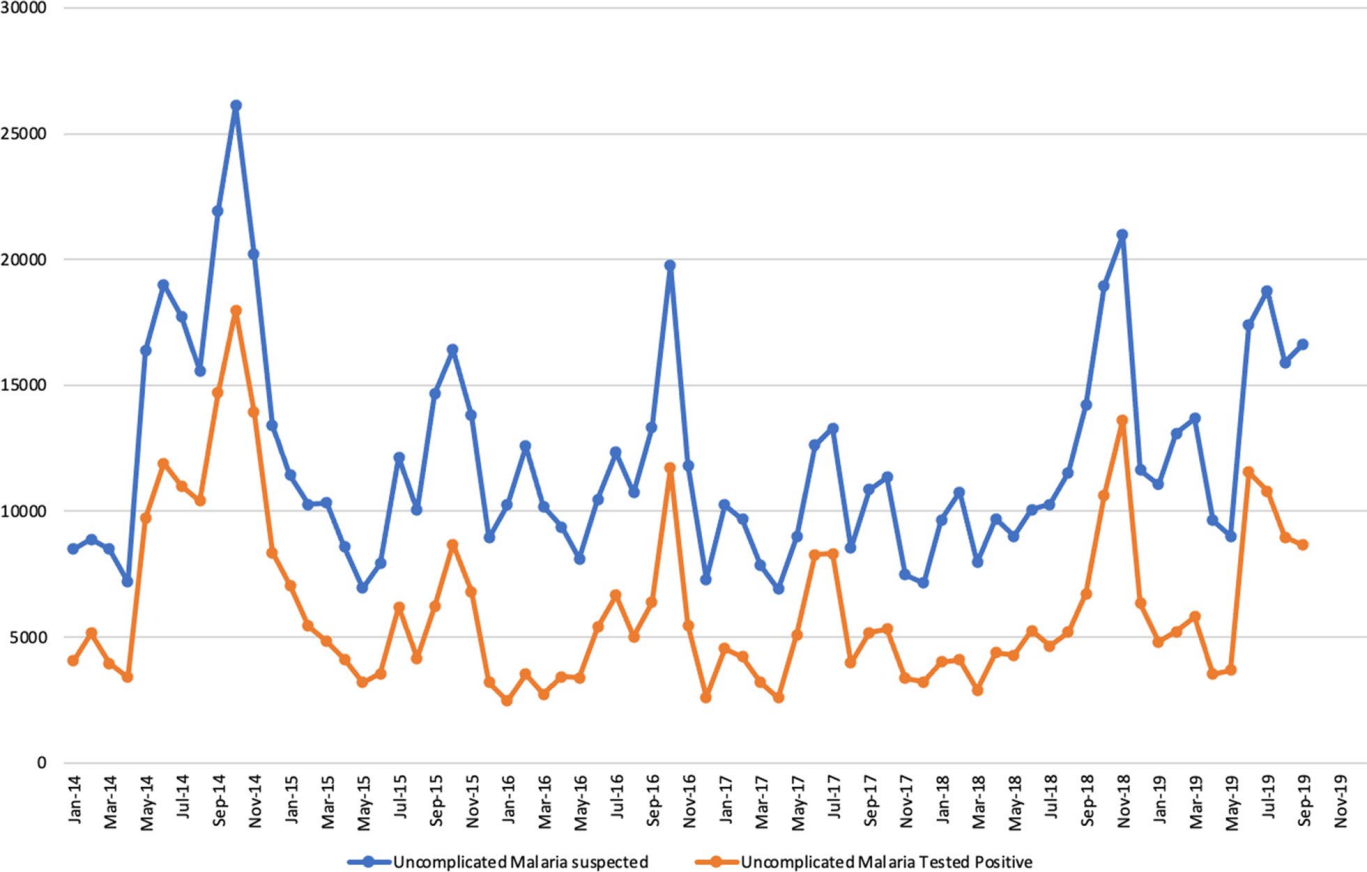
Number of Outpatient malaria cases among children under 5 years in the Upper Westest Region of Ghana; 2014–2019 (Intervention area)

**Fig. 3:**
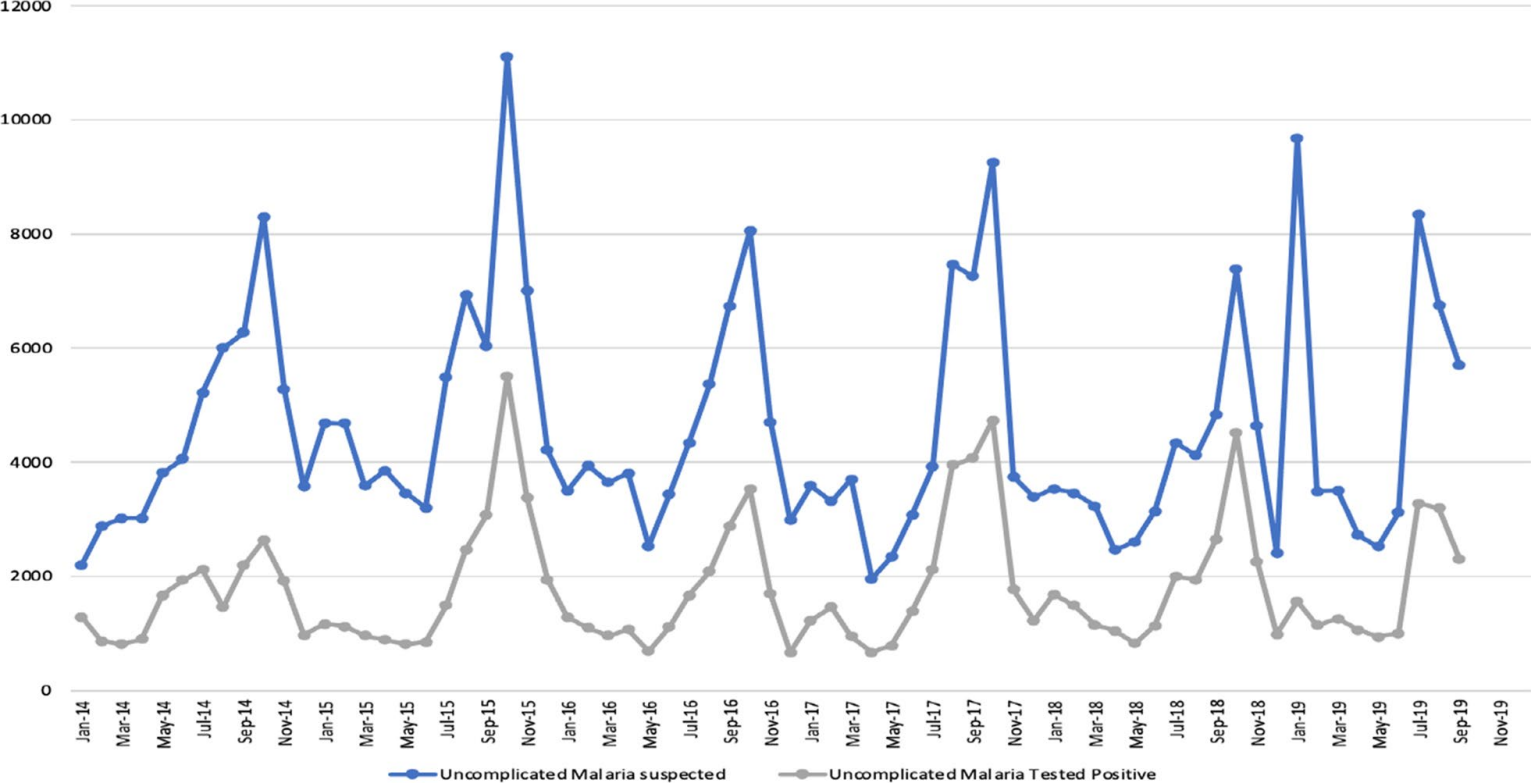
Number of Outpatient malaria cases among children under 5 years in the North East Region of Ghana; 2014–2019 (Non-intervention area)

The evaluation was undertaken by the Navrongo Health Research Centre (NHRC), a field station of the Research and Development Division of the Ghana Health Service [12]. The NHRC runs the Navrongo Health and Demographic Surveillance System (NHDSS), a platform designed to provide efficiency in evaluating health and social interventions [12]. The intervention and the non-intervention took place in two different administrative districts; Lawra district, Upper West Region and West Mamprusi in Northern Region both located in the northern part of Ghana [12–14]. Figure 1 shows the location of the NHRC and the two administrative districts in the respective regions. The study setting is characterized by dryness due to its proximity to the Sahel and Sahara. The vegetation is predominantly Savannah grasslands with clusters of highly drought-resistant trees. There are two main seasons in the area, the dry and wet seasons. The dry season stretches from November to March and the wet season from April to October with a peak in July to September which coincides with malaria transmission. Malaria in northern Ghana is very seasonal [15–17]. Figures 2 and 3 provides historical data from the outpatient departments of the health facilities in the administrative regions that demonstrate seasonal malaria incidence in the two study districts.

In northern Ghana cases of malaria are seasonal caused mainly by *P. falciparum* and transmitted by anopheles’ mosquitoes with *Anopheles gambiae sensu lato* (s.l.) and *Anopheles funestus* being the predominant vector species [15–17].

### Study areas

The study districts have similar seasonal characteristics of malaria cases (Figs. 2 and 3) making them well suited for this study. The intervention area was the Lawra district [13] which lies in the North-Western corner of the Upper West Region of Ghana between latitudes 2^0^ 25"W and 2°45"W and longitudes 10°20” N and 11°00"N. The total population of the area was about 100,929 people. The district had five health centres, ten Community-based Health Planning and Services (CHPS) compounds [18], three pharmacy shops, one clinic and one hospital which provides health services to the people in the area.

The non-intervention area was at the West Mamprusi District [14], one of twenty districts in the Northern Region of Ghana and lies within longitudes 0°35’W and 1°45’W and latitudes 9°55’N and 10°35’N. They had a total population of about 180,877. There was one polyclinic, one health centre, three private clinics, 6 CHPs compounds, and 15 pharmacy shops in this area.

### Study design

The study used a quasi-experimental design with before and after cross-sectional surveys. The implementation was during the main malaria transmission season from July 2015 to the end of the study in December 2015. The duration of participation for each child was six months. Enrolment was conducted over 2 weeks period and the post-implementation evaluation surveys lasted for 2 weeks. The study population included children aged 3 months to 59 months who were eligible for enrolment in the selected households and communities. Two cross-sectional surveys, one at baseline and the other at end line after 6 months were used for evaluation.

The baseline survey took place from July 15, 2015 till July 27, 2015 and first dose was administered in July 27, 2015 and administered for four conservative months at monthly intervals. Continuous monitoring took place from July 27 till December 17, 2015. End line survey was done from December 10, 2015 till December 17, 2015. The enrolled children were followed up between the two surveys for episodes of severe and mild malaria. Other parameters documented included weight, height, malaria parasitaemia, haemoglobin level and temperature.

### Sample size estimation

The sample size calculation was based on the impact of SMC on severe malaria as a primary outcome. Effectiveness assumption is based on studies that showed a reduction in severe malaria by 65 to 86% [6–9]. Using an average proportion of 0.36 of children under 5 years developing severe malaria and 50 children per cluster with an inter-cluster variation of 0.26, approximately 14 clusters were needed per arm to demonstrate a 50% reduction in the proportion of under-five children developing severe malaria at a power of 90% and a two-sided significance level of 5%. A cluster was taken as the community of residence as outlined by the Ghana Health Service District Health Directorate. Each community was a well-delineated area with defined boundaries.

### Sampling strategy

All districts in the Upper West region were listed and one district (Lawra) was selected at random for evaluation of SMC. The control district, West Mamprusi, was selected at random from the Northern Region which had the same routine interventions against malaria as the Upper West Region apart from SMC. All communities in the selected districts were subsequently listed and fourteen out of 141 and 152 communities from Lawra district and West Mamprusi districts respectively were randomly selected. Each selected community was defined as the cluster, the unit of analysis. All the households in each selected community were labelled and listed. All households with eligible children were listed and 40 households randomly selected. In each selected household all eligible children were selected for participation and out of these the children whose guardians gave parental consent and were available for the 4-month duration of the study were selected for inclusion.

A minimum of 50 children per village and 700 children per arm were recruited into the study. Where the community was too small to provide a minimum of 50 participants, the nearest community was added to it to make up a bigger community. Staff visited each household to explain the study in the local language, provided an information sheet, and sought signed consent from parents. Children whose parents consented were enrolled, and mothers/caregivers were issued with a unique identification card bearing the details and study number for each eligible child in their care. The cards were used to identify children if malaria was diagnosed and, for those in clusters randomized to receive SMC, to document SMC courses of treatment received.

### Study drug administration

Study drugs were supplied by National Malaria Control Programme for the regional health directorate. Formulations of sulfadoxine-pyrimethamine 500/25 mg tablet (SP) and amodiaquine (AQ) 153 mg tablet were used. Infants 3- 11 months old were dosed with half of a 153 mg tablet of amodiaquine given once daily for three consecutive days and a single dose of half of 500/25 mg tablet of SP. Children 12–59 months were given a full tablet of 153 mg amodiaquine base given once daily for three consecutive days and a single dose of a full tablet of 500/25 mg tablet of SP. The administration was done by direct observation therapy on the first day and given by community health volunteers. The caregiver gives the rest of the medication on second and third days. This was the intended means of administration by the NMCP. Children received medication for four consecutive months at monthly intervals between July and November 2015.

To determine the proportion of children who experienced an adverse event, study participants in the intervention district were visited at home daily for three days during the administration of SMC and two days after the last dose in each round of drug administration and a side effects questionnaire was completed to document and quantify adverse events that might have occurred since receiving the trial medication. Grading of the severity of adverse events was done by trained field workers. An adverse reaction was graded as mild (grade 1) if it was easily tolerated and moderate to severe (grade 2) if it interfered with normal activity or required treatment.

### Malaria surveillance

A passive surveillance system to monitor malaria episodes was set up at the district hospitals and health centers in the study areas. Fieldworkers were stationed at health facilities and in the intervention and nonintervention districts to pick up any episodes of mild or severe malaria and checked for malaria parasitaemia before treatment was given. Field workers visited the children once a week during the period of drug administration to enquire about their health and completed a morbidity form if a child had any illness. If a child had a history of fever or vomiting within the past 48 h the parents were advised to take their child to the nearest health facility for examination and treatment. Any mortality that occurred outside the health facility was to be investigated and a verbal autopsy questionnaire administered to help ascertain the cause of death at the Navrongo Health Research Centre [12].

All children with fever were encouraged to attend the health post or health centre to be tested with an rapid diagnostic test (RDT) and were treated with artemether-lumefantrine if tested positive. Consultations were recorded in a health facility register to document the test results and treatment given. In the intervention area, if a child was unwell on the day of the SMC visit, caregivers were asked to bring the child to the health post or hospital for testing with malaria RDT and treatment with artemether-lumefantrine if positive. Those who subsequently tested negative received SMC and were referred to the nearest hospital.

Treatment of study participants seen at the health facilities for other conditions was carried out per national guidelines. Registers for recording SMC administration with a list of all children enrolled in each village were made available for each field worker. In SMC villages, the dose of SP and AQ administered to each child was recorded in a register. All consultations for illness were also recorded in the registers and other details including date, symptoms, RDT results and treatment in case report forms. Field workers were recruited and trained to prepare thick and thin films for microscopic examination to crosscheck the RDT results.

### Laboratory methods

Capillary blood was used to prepare two malaria slides for diagnosis. The slides were stained using a 10% Giemsa solution and one examined using x100 oil immersion microscopic lens and the other archived for confirmatory testing. A sample was considered negative only after 200 high power fields had been read. Parasite counts were converted to parasites per microliter (µl), assuming a white blood cell count of 8000 leukocytes per µl of blood. In instances where there were discrepancies in the findings in a slide between the two initial technicians (positive or negative or a 50% or more difference in parasite density), a senior microscopist read the slide and his reading was deemed to be the correct reading. Haemoglobin was measured using Hemocue ® (Hb 801 system).

### Data management and statistical analysis

All consultations for illness were recorded and the register checked for completeness during weekly supervision visits. Completed forms by trained personnel on the study were checked by field supervisors and data managers for consistency and accuracy before logging it out for data entry. All data collected were entered twice into a database using EpiData software. Again, automatic checks for consistency and range errors were done, and queries resolved before the dataset was locked for analysis. Effects of SMC on the prevalence of parasitaemia, gametocyte carriage, mean haemoglobin concentration, and proportions of mild anaemia defined as Hb <110 g/l) and severe anaemia defined as Hb < 60 g/l) were estimated from the survey at the end of the transmission season. The results are in descriptive and analytic statistics and also presented in tables and figures. Analyses were performed using Stata version 14 (Stata Corp, College Station, Texas).

## Results

Baseline characteristics: Seven hundred and thirty-one (731) children were recruited from the intervention district and 708 from the control district. At the end of the intervention, participant retention was 92.9% (697/731) and 89.5% (634/708) in the intervention and the non-intervention areas respectively. Table 1 shows the background characteristics of the children recruited in both districts. Overall, 54.1% of participants were males and most of the children were aged 25 - 59 months (60.1%). The average weight of children at baseline in control district was 11.5 kg (95%CI 11.09, 11.90) and in the intervention district was 11.09 kg ( (95%CI 10.87, 11.32). Again, the average height (in metres) among children at baseline in the control district was 82.71 (95%CI 81.82, 83.61) and 86.93 (95%CI 86.05, 87.81) in the intervention district.

**Table 1:** Background socio-demographic characteristics of the study participants

Attributes	Category	Control district (N = 708)		Intervention district (N = 731)	
		n (%)	(95%CI)	n (%)	(95%CI)
Sex	Male	385 (0.54)	(0.49, 0.59	393 (0.54)	0.49, 0.59
	Female	323 (0.46)	0.41, 0.51	338 (0.46)	0.41, 0.51
Birth order	First	156 (0.22)	0.15, 0.29	202 (27.6)	0.24, 0.36
	Second	204 (0.28)	0.22, 0.34	181 (24.8)	0.19, 0.31
	Third	146 (0.21)	0.14, 0.28	144 (19.7)	0.13, 0.27
	Four or more	202 (0.30)	0.24, 0.36	204 (0.28)	0.22, 0.34
Age groups	3–6 months	61 (0.09)	0.07, 0.11	38 (0.05)	0.04, 0.07
	6–12 months	106 (0.15)	0.12, 0.18	60 (0.08)	0.06, 0.10
	13–24 months	154 (0.22)	0.19, 0.25	155 (0.20)	0.17, 0.23
	25–59 months	387 (0.55)	0.51, 0.59	478 (0.65)	0.61, 0.68
Educational status of care giver	None	649 (0.92)	0.90, 0.94	478 (0.65)	0.61, 0.68
	Primary	34 (0.05)	0.03, 0.07	196 (0.27)	0.24, 0.30
	Secondary	17 (0.02)	0.01, 0.03	39 (0.05)	0.04, 0.07
	Tertiary	8 (0.01)	0.01. 0.02	18 (0.02)	0.01, 0.03
Temp. ≥ 37.5 °C	Yes	10 (0.014)	0.004, 0.020	30 (0.04)	0.03, 0.06
Anti-malarial use in ≤ 2 weeks	Yes	3 (0.004)	0.009, 0.012	26 (0.04)	0.03, 0.06
IRS	Yes	651 (0.92)	0.90, 0.94	720 (0.98)	0.97, 0.99
ITN Use	Yes	493 (0.70)	0.67, 0.73	727 (0.99)	0.98, 1.00
Fever ≤ 2 weeks	Yes	27 (0.04)	0.03, 0.06	40 (0.06)	0.04, 0.08

Overall reported bed net use was almost universal in intervention district (99.5%) as against 69.5% in the non-intervention district. Though indoor residual spraying against malaria was being used by both districts, 57 (8.1%) of the participants in the non-intervention district have not had their house sprayed. For children reporting fever in the week before the survey, 27 (3.8%) of children in non-intervention district reported having fever compared with 40 (5.5%) % of children in the intervention district. However, 30 (4.1%) of children in intervention district compared with 10 (1.1%) in the non-intervention district had temperature ≥37.5ºC at the time of the enrolment. The use of anti-malarial two weeks before the survey was higher 26 (3.6%) in intervention district compared with 3 (0.4%) in non-intervention district (Table 1).

Incidence rates of confirmed severe and uncomplicated malaria: A total of nine (9) cases of severe malaria were recorded in the two districts. There were three (3) cases in the intervention area and six (6) in the non-intervention area including one (1) death. The incidence rates of severe disease in the intervention district were 10 per 1000 person-years at risk as against 20 per 1000 person-years at risk in the non-intervention district with an adjusted rate ratio of intervention to non-intervention district of 0.52 giving protective effectiveness of 48% (p = 0.62) against malaria (Table 2). For clinically diagnosed malaria with any level of parasitaemia, the incidence rates were 220 and 170 per 1000 person-years at risk for intervention and non-intervention areas, respectively (Table 2). There was only one death in the study due to severe malaria and it occurred in the non-intervention district.

**Table 2:** Impact of seasonal malaria chemoprevention on clinical malaria

Outcomes	Intervention area		Nonintervention area		Unadjusted IRR	Adjusted IRR	PE (95% CI)	P-value
	n episodes (years at risk)	Incidence rate (95% CI)	n episodes years at risk	Incidence rate (95% CI)				
Malaria diagnosis with any asexual parasitaemia	55 (241.53)	0.22 (0.17- 0.30)	42 (243.69)	0.17 (0.13- 0.23)	1.29 (0.83- 1.99)	1.25 (0.81- 1.93)	− 25	0.31
Malaria diagnosis with parasitaemia>=5000	46 (241.53)	0.19 (0.14- 0.25)	31 (243.69)	0.12 (0.09-0.18)	1.48 (0.92- 2.38)	1.42 (0.88- 2.30)	− 42	0.15
Severe malaria	3 (307.2)	0.01	6 (285)	0.02	0.45	0.52 (0.04- 7.04)	48	0.62

Effect of SMC on parasitaemia (Table 3): The proportion of children with parasites increased significantly from 9.0% (95% CI 7, 11) at baseline to 21.5% (95%CI 18, 24) at the end-line in the non-intervention district (P < 0.0001). In the intervention district however, the proportion of children carrying asexual parasites reduced significantly from 31.1% (95%CI 28, 36) to 12.1% (95%CI 10, 15), a difference of 19% (95%CI 15, 23) p < 0. 000.1 (Table 3).

**Table 3:** Comparison of baseline and endline indices of the study

Variable	Study area	Baseline	End line	95% CI	P-value
Children with Parasitaemia, n (%)	Intervention	227 (31.1)	82 (12.1)	19 (14.8,23.1)	0.000
	Non-intervention	64 (9.0)	136 (21.5)	− 12.5 (− 16.3, − 8.6)	0.000
Children with Gametocytes, n (%)	Intervention	13 (1.8)	22 (3.2)	− 1.4 (− 3.0, -0.2)	0.090
	Non-intervention	4 (0.6)	35 (5.5)	− 4.9 (− 6.7, − 3.0)	0.000
Children with Hb <11.0 g/dl, n (%)	Intervention	386 (52.8)	262 (38.6)	14.2 (9.0,19.3)	0.000
	Non-intervention	386 (54.5)	397 (62.6)	− 8.1 (− 13.3, − 2.8)	0.003
Children with Hb < 8.0 g/dl, n (%)	Intervention	26 (4)	11 (2)	2 (0.2, 3.7)	0.028
	Non-intervention	27 (4)	41 (6)	− 2.0 (− 4.3, 0.34)	0.091

		Baseline		Endline	
		Mean	95%CI	Mean	95%CI
Mean Parasite Density (asexual)	Intervention	1883	1424, 2489	3349	2198, 5103
	Non-intervention	370	219, 626	1454	1053, 2009
Mean Parasite Density (sexual)	Intervention	75	44, 127	209	133,330
	Non-intervention	204	36,1137	123	85,180
Total Mean Haemoglobin (Hb)	Intervention	10.79	10.69,10.88	11.18	11.07, 11.31
	Non-intervention	10.77	10.66,10.88	10.53	10.41, 10.66

At baseline, the geometric mean trophozoite and gametocyte counts for intervention district were 1883 parasites/dl and 75 parasite/dl, respectively. Both increased at the end-line to 3349 parasites /dl and 209 parasite/dl, respectively. The number of children carrying gametocytes almost doubled (from 13 (1.8%) to 22 (3.2%) at the end of the SMC administration.

In the non-intervention district, the geometric mean trophozoite and gametocyte counts were 370 parasites/dl and 204 parasites/dl respectively at baseline and 1452 parasites/dl and 123 parasites/dl at endline. The number of children carrying gametocytes was 4 (0.6%) at baseline and 35 (5.5%) at endline.

### Effect of SMC on haemoglobin (Hb) levels (Table 3)

The mean Hb level of participants at baseline in the intervention area were 10.79 g/dl (95%CI 10.69, 10.88) and this increased to 11.18 g/dl (95%CI 11.07, 11.31) at the end line. For the non-intervention area, the mean Hb levels of participants reduced from 10.77 g/dl at baseline to 10.53 g/dl at endline. For all participants present at the end line, there was a mean reduction in Hb levels of 0.24 g/dl for participants at the non-intervention district and increase of 0.39 g/dl for participants from the intervention district and both were significant (p < 0.0001).

The proportion of children with Hb < 11.0 g/dl and Hb < 8.0 g/dl at baseline was 52.8% (95%CI 51, 58) and 4% (95% CI 3, 5) respectively in the intervention district. At the end line, the proportion of children anemic with Hb less than 11.0 g/dl and 8.0 g/dl reduced to 38.6% (95%CI 35, 43) and 2%% (95%CI 1,3) respectively and both were statistically significant (P < 0.0001). In the non-intervention district however, both proportions increased from 54.5 to 62.6% in those with Hb <11/0 g/dl and from 4 to 6% in those HB <8 g/dl and were also statistically significant (P < 0.0001).

### Adverse events experienced by participants following SMC administration (Table 4)

**Table 4:** Frequencies of Reported Adverse Event by Participants per Treatment Round

Adverse events	Number per Treatment Round				
	One (N = 731)	Two (N = 635)	Three (N = 587)	Four (N = 621)	Total
Vomiting	33	27	33	51	144
Fever	7	19	32	27	85
Diarrhoea	26	25	7	12	70
Weakness	11	7	3	3	24
Abdominal pains	0	5	0	0	5
Cough	0	14	0	5	19
Rashes	0	9	4	0	13
Others	6	3	13	14	36
Total	83	109	92	112	396

**Fig. 4:**
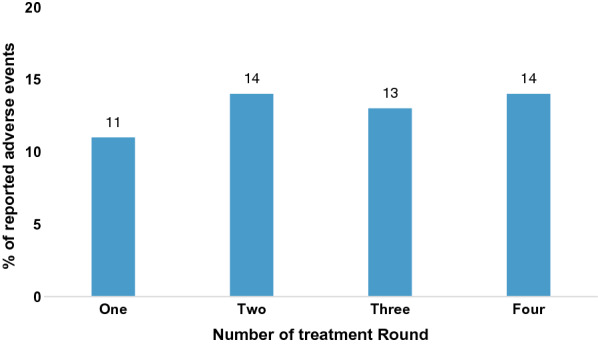
Percentage of children experiencing at least one adverse event per treatment round

Of the 731 children recruited in the intervention district, 635 (87%) received the second dose, while 587 (80%) received the third dose. In the last round, 621 (85%) took the intervention drug. Most participants received the intervention except those who had travelled or had taken prior malaria treatment. There was no severe adverse event associated with the drug administration. At least 11% of children experienced one adverse event or the other during each round of administration (Fig. 4). Diarrhoea, vomiting, and fever were the most experienced adverse events during the four rounds of SMC administration. Most of the adverse events were malaria-like symptoms and were mild to moderate and all resolved within the week. The frequencies of adverse events experienced are summarized in Table 4.

## Discussion

Several studies have demonstrated the efficacy of SMC under varying health systems (3,6,7), however, the feasibility and potential challenges of scaling up the SMC in northern Ghana needed to be assessed under the existing public health system. The study documented how SMC as a malaria control tool could be operationally initiated and scale up in the most efficient and effective way in Ghana. This is because in Ghana, the health system depends on a complex interplay of factors including the local community dynamics, drug acceptability, climate conditions, malaria transmission dynamics and population distribution. This pilot to document the effectiveness and best practices for SMC implementation in Ghana was undertaken.

From the study results, the overall participant retention rate was very satisfactory and consistent with previous SMC studies [3]. High participant retention is important as it serves as a proxy of how effective a programme will be if it is fully scale up in normal conditions. High dropouts in programme interventions can engender staff apathy, increase implementation costs and longer duration of implementation among others. In addition, this study gave an indication on the optimal staff numbers needed for scale up and how community health volunteers could be used to implement SMC in Ghana. Parental cooperation and appreciation of the intervention are suggestive of the potential acceptance of SMC in these settings. These were further boosted by the reported adverse events which were mild and consistent with routine malaria symptoms ranging from 10 to 15%.

The results also give an indication on how SMC could impact on malaria outcomes. From the results though the number of severe outcomes captured in this study was low, having six severe outcomes including death in the non-intervention compared to three in the intervention arm is an indication of potential good impact. While the difference was not statistically significant probably due to the small numbers and the sample size, as a pilot study this gives an indication of how effective the SMC intervention could be in our settings when the actual programme is implemented and scaled up. Moreover, the rate of 10 per 1000 child years at risk in the intervention arm is half that of the nonintervention group and consistent with other low rate reported elsewhere in Mali [7]. There were about 20% reduction in the proportion of children carrying parasites in the intervention areas compared to about 12% in increase in the nonintervention area. This is relevant as chronic malaria infection are known to be the major cause of anaemia in children in endemic areas. The results did not show any impact of SMC on sexual malaria parasite suggestive the drugs used may not be able to suppress transmission in the short term.

The proportion of gametocytaemia increased in both districts and the increase at the endline was much higher in the non-intervention group. This suggests that the drug combination at this present moment is not effective in killing the sexual forms of the parasite responsible for malaria transmission.

This notwithstanding, at the end of the transmission season, moderate anaemia was reduced by about 50% in the intervention arm which was consistent with earlier studies [3–8]. This was in contrast with an increased proportion of about 25% in the non-intervention area. A similar effect was documented with mild anaemia. This is noteworthy because despite the high intensity of malaria infection in the intervention area, children were protected against anaemia. Moreover, children were able to build up their haemoglobin levels as clinical and subclinical malaria parasitaemia is reduced. SMC therefore even without iron supplementation would be a good tool for the prevention of anaemia due to malaria in these children.

The safety and tolerability of SMC in the intervention area were good. There were no serious adverse events reported immediately following SMC administration and there was no report of stopping subsequent administrations due to safety of the drugs. Though severe malaria and a death were recorded these were not attributable to the drugs.

Diarrhoea and vomiting were the dominant adverse drug reactions, but they were mostly self-limiting. At least 11% of children taking the SMC medications at a time experienced adverse drug reactions and this is much higher than reported in other studies. The level of adverse drug reactions reported in this study is thus acceptable especially as they were all self-limiting and all resolved without sequelae. The study has been limited by the sample size and number of study sites.

## Conclusions

The pilot study showed SMC provided substantial protection against anaemia and malaria parasite carriage in children in the Northern Sahelian belt of Ghana in addition to the other control measures like IRS and ITN use. The choice of amodiaquine and sulfadoxine-pyrimethamine is safe and tolerable among children in this region, though a drug with gametocidal effects in the future will provide additional effects in clearing gametocytes at the end of season. These findings support the proposal to scale up the implementation of SMC to the other two regions of northern Ghana.

## Data Availability

The datasets during and/or analysed during the current study available from the corresponding author on reasonable request.

## References

[1] WHO. World Malaria Report 2020. Geneva, World Health Organization, 2020. https://www.who.int/docs/default-source/malaria/world-malaria-reports/9789240015791.

[2] WHO. Global Technical Strategy for Malaria 2016–2030. Geneva, World Health Organization, 2015. https://www.who.int/malaria/publications/atoz/9789241564991/en/.

[3] Wilson AL (2011). A systematic review and meta-analysis of the efficacy and safety of intermittent Preventive treatment of malaria in children (IPTc). PLoS One.

[4] WHO (2013). Seasonal malaria chemoprevention with sulphadoxine-pyrimethamine plus amodiaquine in children: a field guide.

[5] WHO. Report of the technical consultation on seasonal malaria chemoprevention (SMC Geneva, World Health Organization, 2011. http://www.who.int/malaria/publications/atoz/smc_report_teg_meetingmay2011.pdf.

[6] Cairns M, Roca-Feltrer A, Garske T, Wilson A, Diallo D, Milligan P (2012). Estimating the potential public health impact of seasonal malaria chemoprevention in African children. Nat Commun.

[7] Dicko A, Diallo AI, Tembine I, Dicko Y, Dara N, Sidibe Y (2011). Intermittent preventive treatment of malaria provides substantial protection against malaria in children already protected by an insecticide-treated bednet in Mali: a randomized, double-blind, placebo-controlled trial. PLoS Med.

[8] Coldiron ME, von Seidlein L, Grais RF (2017). Seasonal malaria chemoprevention: successes and missed opportunities. Malar J.

[9] Sokhna C, Cisse B, Hadj Bâ E, Milligan P, Hallett R, Sutherland C (2008). A trial of the efficacy, safety and impact on drug resistance of four drug regimens for seasonal intermittent preventive treatment in Senegalese children. PLoS One.

[10] Onyango EO, Ayodo G, Watsierah CA, Were T, Okumu W, Anyona SB (2012). Factors associated with non-adherence to artemisinin-based combination therapy (ACT) to malaria in a rural population from holoendemic region of western Kenya. BMC Infect Dis.

[11] Annual Situational Report 2014. National Malaria Control Programme (NMCP), Ghana Health Service. Ministry of Health Ghana.

[12] Oduro AR, Wak G, Azongo D, Debpuur C, Wontuo P, Kondayire F (2012). Profile of the Navrongo Health and Demographic Surveillance System. Int J Epidemiol.

[13] Ghana Statistical Service (2014). District Analytical Report. Lawra district

[14] Ghana Statistical Service (2014). District Analytical Report. West Mamprusi district

[15] Koram KA, Owusu-Agyei S, Fryauff DJ, Anto F, Atuguba F, Hodgson A (2003). Seasonal profiles of malaria infection, anaemia, and bednet use among age groups and communities in northern Ghana. Trop Med Int Health.

[16] Oduro AR, Koram KA, Rogers W, Atuguba F, Ansah P, Anyorigiya T (2007). Severe falciparum malaria in young children of the Kassena-Nankana district of northern Ghana. Malar J.

[17] Appawu M, Owusu-Agyei S, Dadzie S, Asoala V, Anto F, Koram K (2004). Malaria transmission dynamics at a site in northern Ghana proposed for testing malaria vaccines. Trop Med Int Health.

[18] Nyonator FK, Awoonor-Williams JK, Phillips JF, Jones TC, Miller RA (2005). The Ghana community-based health planning and services initiative for scaling up service delivery innovation. Health Policy Plan.

